# Deprescribing of Medicines in Care Homes—A Five-Year Evaluation of Primary Care Pharmacist Practices

**DOI:** 10.3390/pharmacy7030105

**Published:** 2019-08-03

**Authors:** Ana Alves, Shaun Green, Delyth H James

**Affiliations:** 1 NHS Somerset Clinical Commissioning Group, Yeovil BA22 8HR, UK; 2 Department of Applied Psychology, Cardiff School of Sport and Health Sciences, Cardiff Metropolitan University, Llandaf Campus, Cardiff CF5 2YB, UK

**Keywords:** care homes, deprescribing, pharmacists

## Abstract

(1) Background: This project evaluates the outcomes of a novel pharmacy-led model of deprescribing unnecessary medications for care home patients. A feasibility study was conducted in 2015 to explore exposure to inappropriate polypharmacy in patients residing in care homes over a one-year timescale. The aim of this study was to present the results of this ongoing service evaluation over a five-year period. (2) Methods: Data collection and risk assessment tools developed during the feasibility study were used to measure the prevalence, nature, and impact of deprescribing interventions by primary care pharmacists over a five-year period. A random sample of approximately 5% of safety interventions were screened twice yearly by the pharmacist leads as part of standard practice. (3) Results: Over a period of five years there were 23,955 interventions (mean 2.3 per patient) reported from the 10,405 patient reviews undertaken. Deprescribing accounted for 53% of total estimated financial drug savings, equating to £431,493; and 16.1% of all interventions were related to safety. (4) Conclusions: Medication reviews in care homes, undertaken by primary care pharmacists who are linked to GP practices, generate a wide range of interventions commonly involving deprescribing, which contributes significantly to the continuous optimisation of the prescribing and monitoring of medicines.

## 1. Introduction

### 1.1. Overview

Appropriateness, safety, and quality of prescribing are concepts closely linked to the overall well-being of the frail, elderly patients living in care homes who often take multiple medicines and have complex needs [1]. This study focuses on a model of medication optimisation reviews undertaken by primary care pharmacists working with General Practitioners (GPs) in Somerset, which led to a range of clinical recommendations in relation to drug prescribing and monitoring. This paper presents the findings of the research specifically related to deprescribing. The following sections provide further details on the background and considerations which led to selecting the aim of the present study.

### 1.2. Prescribing and Polypharmacy

Prescribing of medicines to patients is recognised as the most common healthcare intervention in the National Health System (NHS), representing its highest expenditure (£8.8 billion in 2018) immediately after staffing costs [2,3]. The vast majority of NHS prescription items are issued by GPs in primary care. In England, there has been an increasing trend in prescribing [3] driven by a combination of numerous factors. These include population growth, higher proportion of the older population, increase in diagnosis capability, and availability of new medicines to prevent or reduce the risk of potential health problems. These factors have also contributed to the rise of a phenomenon defined in clinical practice and known as *polypharmacy*, particularly occurring in debilitated populations such as the frail elderly and those with co-morbidities, whereby patients receive a combination of several medicines. These are common features of a significant proportion of patients residing in nursing and residential homes [4].

Taking multiple medicines is deemed beneficial if patients are adherent to this approach, tolerate such combinations well, and the therapy is appropriate to treat their medical conditions, with the ultimate goal of maintaining or improving quality of life. However, polypharmacy is considered inappropriate if the medication is clinically unnecessary, in cases where patients do not benefit from the prescribed therapy as intended, or the risks of harm from taking combined medicines are higher than the potential benefits; it can lead to intolerable side-effects, difficulty in taking medication, and adverse interactions between drugs [5].

### 1.3. Medicines Optimisation and Deprescribing

Deprescribing has been the subject of growing interest in the scientific research community in recent years [6,7,8,9,10,11,12]. Deprescribing is a complex process required for the safe and effective cessation (withdrawal) of inappropriate medication. It considers the patient’s physical functioning, co-morbidities, preferences, and lifestyle. Despite limited established guidance and evidence surrounding its principles and outcomes, the emerging research suggests that appropriate deprescribing, especially for older patients with complex regimens, is generally safe, and patients are receptive to it [13,14,15,16,17,18,19].

A systematic review in 2008 [20] investigated clinical trials undertaken since 1966 which included withdrawal of specific classes of medicines in patients aged 65 years and over. The authors found 31 relevant studies with almost 9000 patients and showed evidence of benefits and safety in the short-term of stopping psychotropics, antihypertensives, and benzodiazepines. Another systematic review [21], published in 2014, found an association between deprescribing and improvement of clinical management of falls and cognitive function, which are recognised as significant morbidity and mortality factors. A more recent systematic review [22] of deprescribing interventions in hospitalised patients found that stopping inappropriate medications is feasible, effective, and safe. However, to date there have been no systematic reviews of the literature of deprescribing in care home settings.

### 1.4. Medicines Interventions in Care Homes

The “Care homes’ use of medicines study (CHUMS)” [23] highlighted the need for and the benefits of pharmacists’ interventions in patient care, medicines optimisation, and safety. It found that the 256 participants from 55 care homes were on average prescribed 7.2 medicines each. It also estimated that on any given day 70% of patients were subject to at least one medication error associated with an adverse event caused by poor clinical management such as monitoring (14.7%), dispensing (9.8%), administration (8.4%), and prescribing (8.3%). The authors concluded that care home patients were exposed to an unacceptable level of medication related errors. This paper called for action from all involved and has also influenced subsequent national guidance in the United Kingdom (UK).

A recent cohort study [24] concluded that polypharmacy is common in nursing home residents in Europe and decisions to deprescribe are dependent on individual and organisational factors such as clinical conditions and staff involved.

### 1.5. Study Setting

The most significant structural reform in recent years in the NHS in England was the establishment of Clinical Commissioning Groups (CCGs) in April 2013. Each one of these clinically-led organisations is representative of all GPs in a given geographical area. CCGs have the responsibility of contracting most community and hospital health care services, including prescribing for local patients on behalf of and overseen by NHS England (NHSE). The NHS Somerset CCG Medicines Management Team has historically and to varying degrees facilitated medicines optimisation reviews for patients living in care homes, as a way to reach out to patients who are predominantly frail, elderly, and living with co-morbidities, as well as providing support for carers. In Somerset, in 2014, a novel model was developed in order to standardise medication optimisation reviews to patients in care homes. At the time there was emerging evidence indicating that there was a growing need for establishing this type of service.

As part of this model of care, a team of primary care pharmacists, funded by NHS Somerset CCG supported GP practices in undertaking medication optimisation reviews in care homes. The aim of the service was to address inappropriate polypharmacy and prevent drug-related harm, utilising primary care pharmacists in liaison with GPs. A feasibility study was conducted in 2015 to evaluate a number of aspects surrounding this model of deprescribing of medicines in a sample of care homes in Somerset [25]. As a result, the Clinical Operations Group at NHS Somerset CCG recognised the benefits to patients and the local healthcare system by agreeing to grant recurrent and ongoing funding for this novel model of care. Likewise, nationwide investment in the form of support to care homes was recently launched by NHSE as “Medicines Optimisation in Care Homes (MOCH)” with the purpose of improving the safety and quality of care for people in care homes [26]. The MOCH service is currently commissioned through NHS Somerset CCG to local secondary care providers, and is based on the original Somerset model of care; funding is expected to be transferred to the emerging Primary Care Networks [27].

This project evaluates the outcomes of this novel pharmacy-led model of deprescribing of unnecessary medications for care home patients. The 2015 feasibility study to explore exposure to inappropriate polypharmacy in patients residing in care homes was conducted over a one-year timescale [25]. The purpose of this paper is to present the results of this ongoing service evaluation over a five-year period.

### 1.6. Aim

The aim of this research was to evaluate the prevalence and nature of interventions, the rationale for stopping medicines, and the impact of deprescribing on patient safety in care homes over a five-year period.

## 2. Materials and Methods

### 2.1. Overview of Study

The study adopted a mixture of quantitative and qualitative methodology in order to address the aims of the study. Data collection and risk assessment tools were developed during a feasibility study undertaken in 2015 [25]. These were used to measure the prevalence, nature and impact of deprescribing interventions by primary care pharmacists over a five-year period. The number of deprescribing interventions that occurred were quantified within eight distinct categories, and the financial costs were calculated over a period of five years. A safety risk assessment took place for all interventions, and further case studies were described to illustrate the nature of these.

### 2.2. Approvals and Ethical Considerations

Under the NHS Healthcare Research Authority [28] standards and definition of research, the feasibility project from which this present study builds upon, was formally classified as a *service evaluation* and therefore did not require submission to the NHS Research and Development or ethical review by a NHS Research Ethics Committee.

### 2.3. Model of Service Delivery

The service was designed by two of the authors (A.A. and S.G.) in order to develop a standardised and consistent approach across the county of Somerset. The model of service delivery was based on the skill set of the existing pharmacy staff at the time, in addition to critical incidents and feedback from a variety of stakeholders (care homes’ staff, GP practices, and primary care pharmacists). Considering Somerset’s wide rural geography, the aim of the programme was to offer at least one medicines optimisation visit to as many care homes (residential, nursing and mixed) as possible, by primary care pharmacists on behalf of GP practices.

The time and level of support allocated for the service was agreed upon with the respective CCG Locality Pharmacist Manager and influenced by a number of factors such as engagement from GP practices; primary care pharmacists’ availability; skills and confidence; number of care home patients registered with each GP practice; and geographic area covered by the prescribing support pharmacists. The type of service provided was to some extent dependent on the degree of involvement from other healthcare professionals, such as whether a GP visited the care home alongside the pharmacist, or if pharmacists undertook the review and had a prior discussion with the prescriber at the GP practice.

#### Quality Assurance

The CCG Locality Medicines Managers reviewed all patient-anonymized care home reports submitted by the primary care pharmacists; and twice yearly the CCG pharmacist project lead (A.A.) undertook a review of a sample of approximately 5% of safety interventions with the team colleague who leads on safety. This is standard practice and considered to be an effective mechanism of providing feedback to the team. Good practice, learnings and relevant observations are widely shared with the primary care community through regular email updates, Learning Engagement Network meetings, and GP Prescribing Leads events.

### 2.4. Data Collection Tools

Standardised data collection tools used by the pharmacists were developed as part of the feasibility study conducted in 2015 [25]. The primary care pharmacists continued to use these forms for 2015–16, 2016–17, 2017–18, and 2018–19 as part of care home visits, which the CCG used to collate overall information on interventions. These consisted of the following:

- Form 1: Care Home Data Collection Form (Table S1),
- Form 2: Care Home Intervention Codes (Table S2),
- Form 3: Care Home Safety Risk Assessment Scoring (Table S3), and
- Form 4: Care Home Visit Flowchart (Figure S1).

Forms 1,2, and 4 were developed in conjunction with the pharmacy team which involved several iterations to produce the most effective version to capture the interventions in an efficient manner.

#### Development of Safety Risk Assessment Scoring Scale

The safety risk assessment scoring scale (Form 3) was developed locally [25] based on the findings from well recognised studies [29,30] and utilised consensus methodology. The first phase of the process consisted of a peer-consensus from two CCG lead pharmacists. Phase 2 involved a clinical validation panel (two GPs, one hospital and one integrated-services pharmacist), and the third phase comprised expert review by one care of the elderly consultant [25]. A number of changes were made to the scale between each phase until 100% agreement was reached from all parties.

### 2.5. Study Procedures

All intervention data were captured on Form 1 by the pharmacist conducting the intervention, using Forms 2 and 3 to code the data. Likewise, the pharmacists also calculated the estimate drug saving by referring to the most recent edition of the Drug Tariff to find the price of the drug. All forms were anonymised before being sent from the care home. Completed Forms 1 were received electronically by the CCG Medicines Management team, and the administrator merged the information with an overall data Excel spreadsheet (Microsoft Office 2010 version 16.0) populated with a macro.

### 2.6. Analysis

The data were extracted from the Excel spreadsheet containing information from each financial year and overall five years. The quantitative information included the number of care homes reviewed, number of patient reviews, time spent on review, number of interventions and type, deprescribing category, safety risk level, age of patient, total number of drugs, potential annual drug savings, and savings from deprescribing. The 28-day cost of the drug was multiplied by thirteen to obtain an annual estimation of the cost saving. Pharmacists’ time was estimated based on an hourly rate of £26. Overall cost savings were calculated by subtracting the pharmacists’ time away from the overall drug cost saving.

The descriptive data were extracted for individual patients to illustrate the nature of the intervention, actions taken by the care home and/or GP practice, and outcomes from safety interventions recorded.

## 3. Results

In the period between April 2018 and March 2019, of the 27 pharmacists commissioned by NHS Somerset CCG, 25 conducted care home reviews which were included in the service Level Agreement (between 4 and 12 h per week). In comparison, at the start of the model of delivery in 2014–15, 12 pharmacists provided this service.

As an example of one-year’s output, Table 1 summarises the results for 2018–19 across GP practices grouped into seven geographical areas (known as GP Federations). There have been 2927 patient reviews across 121 nursing and residential care homes of which 17 received a second visit. The overall spend in pharmacist time was £63,444, equating to £21.68 per patient review.

Table 2 presents the total type of interventions, as well as those coded as potentially having an impact on patient safety, and the nature of deprescribing interventions. Data for the 2018–19 year shows that a total of 5168 interventions were recorded for 2927 patient reviews, of which deprescribing represents approximately one quarter. Although a small proportion, there were also 86 opportunities identified to initiate appropriate medication. The overall savings were £148,656 when subtracting the cost associated with pharmacists’ time.

Details of safety related interventions were also collated, demonstrating that most of the 1143 identified as part of the reviews were deemed *minor* or *moderate/significant* (combined 98.7%). It was expected that these patients would have been likely to experience increased side-effects and need additional monitoring had the pharmacists not intervened. Thirteen interventions were classed as major/serious, indicating that the probability of admission was high had these not occurred. Moreover, for the first time since the start of the project, there were two interventions classed as *Catastrophic/Potentially Lethal*, meaning that if the interventions had not taken place, hospital admission would have been certain. On these two occasions the errors were linked to unintended prescribing of a high-risk drug. In the first case a Direct Oral Anticoagulant (DOAC) was initiated for the incorrect patient for whom there was no indication of such a drug. In the second case, the patient had been admitted to hospital with hypoglycaemia but the dose of a sulphonylurea was doubled in the care home instead of halved after discharge from hospital. Pharmacists reported that the most common reason for deprescribing was that drugs were no longer required by the patients (59.3% of deprescribing interventions).

**Figure 1:**
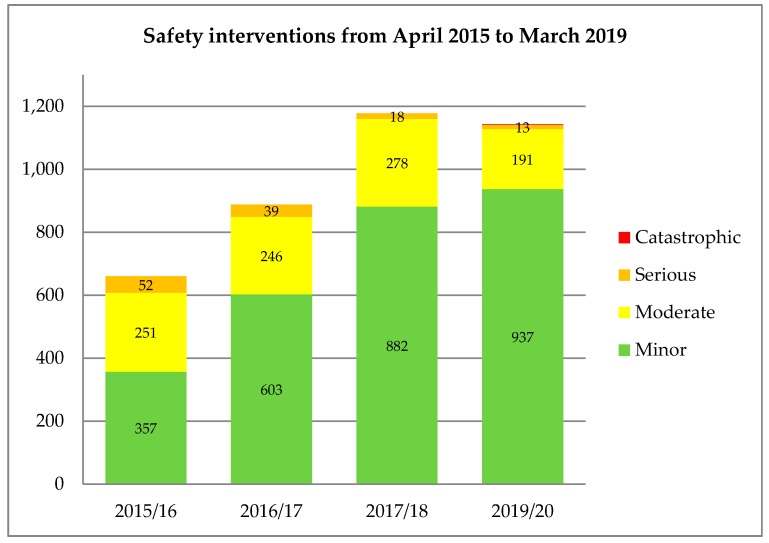
Safety interventions by category and financial year.

The lead pharmacist for the programme delivery (A.A.) and the colleague responsible for safety across the organization (S.G.) carried out a joint review of a sample of safety interventions twice yearly. The sample comprised the last 30 to 40 interventions collated from the last entries for the period being reviewed until at least three examples of each risk level had been identified (all interventions scored as level 4 are always independently reviewed by two people). An extract for the latest sample of interventions reviewed is provided in Table 3:

Table 4 provides an overview of the progress over five years from baseline in 2014–15 up until 2018–19. This demonstrates that 10,405 patient reviews led to an average of 2.3 interventions per patient and just over a sixth of all interventions were reported as having a potential impact on patient safety (16.1%). Over time the estimated potential annual drug savings was approximately £812k with savings per patient review gradually reduced from £89 to £78. Overall, the savings from deprescribing over five years amounted to £431,483 (53% of total savings):

Figure 1 presents the number of safety interventions identified each year by the primary care pharmacists. The main changes have been an increase in the overall number of *minor* safety interventions reported, and a reduction in the proportion of those categorised as *serious/moderate*, with the only two catastrophic/potentially lethal identified in 2018–19.

## 4. Discussion

The aim of this study was to explore the impact of deprescribing interventions over a five-year period. In summary, there were nearly 24,000 interventions reported across the 25 pharmacists delivering the scheme, who undertook over 10,000 patient reviews. This equated to an average of over two interventions per patient. Deprescribing accounted for over half of the total estimated financial drug savings, equating to nearly half a million pounds. Approximately one-sixth of all interventions were related to safety.

These findings suggest that the Somerset model of pharmacist deprescribing in care homes has an important impact on patient care in terms of increasing appropriate prescribing, enhancing safety and producing significant cost savings for the NHS. The data generated since the feasibility study [25] was conducted demonstrate that deprescribing interventions by pharmacists in care homes provided a consistent and sustainable approach over 5 years.

Since the service was first initiated in 2014, the NHS Somerset CCG programme has expanded considerably and in recent years pharmacists reached out to more than twice the number of patients and care homes when compared to the start of the scheme. Medicines optimisation in care homes have become embedded in the NHS Somerset CCG medicines management workplan and part of the core prescribing support service to GP practices. The results gathered show that the main opportunities were in deprescribing unnecessary medicines and medication safety; pertinently, both themes are under close national attention as part of the national health and care agenda. Whilst contributing to the safety agenda, deprescribing also consistently remained the most significant intervention in terms of releasing significant estimated financial savings for an increasingly constrained healthcare budget. Over the period of five years, it equated to over £431k (53%) from a total savings of £812,441.

The evidence base surrounding deprescribing continues to grow, and despite the lack of systematic reviews specifically concerning the care homes setting, a recent randomised controlled trial [29] in 59 Dutch nursing homes demonstrated that deprescribing can be effectively and safely achieved in a significant proportion of residents (29.5% to 39.1%). A Cochrane review updated in 2016 [30] despite being inconclusive on the impact on clinical and safety aspects from medication optimisation in care homes, highlights that evidence was found in relation to solving medication problems.

In this study, sub-analysis of the most recent financial year (2018–19) revealed that most deprescribing interventions as part of 138 care home visits were due to the lack of a need for medicines (59.3%), contributing to a reduction in unnecessary polypharmacy. These findings resonate with a recent systematic review and meta-analysis by Hansen et al. [31]. The research concluded that deprescribing can be successfully achieved in association with behaviour change techniques, resulting in a decrease in medications and inappropriate prescribing in older people. A limitation of this research lies in the fact that formal and systematic economic analysis has not been performed as part of this evaluation; nevertheless, available data shows that it delivers potential considerable drug savings, with a 2.34 times equivalent return on investment in pharmacist salary time in 2018–19. This is without factoring in the potential improved outcomes and reduced NHS and social care activity from the scheme. Another limitation remains in the tool adapted from the Medicines Management team from the Enhancing Quality of Life in Patients (EQUIP) and Discharge Medication Review (DMR) studies [32,33], which was not fully validated and therefore it is unclear whether it can be adequately and accurately applied to all types of interventions. However, findings from the feasibility study [25] were still observed in that reducing the burden of polypharmacy for patients in care homes remained an important aspect of effective prescribing support provided by primary care pharmacists, and contributed to a patient-centred approach to their safety and care.

Nonetheless, the resources developed to record and analyse data from medication reviews in care homes have already been shared with neighbouring CCGs and through the national Future Learn platform [34] dedicated section for the NHS England MOCH programme. With the imminent formation of Primary Care Networks [35] it is hoped that a structured programme will be developed for primary care clinical pharmacists to routinely undertake medication reviews for care home patients. It is recommended that consideration is given from this five-year evaluation which signals that a centralised model of management covering the whole county has been instrumental in providing a structured approach, as well as facilitating a platform for skill development and sharing of best practice.

## 5. Conclusions

Medication reviews in care homes, undertaken by primary care pharmacists who are linked to GP practices, generate a wide range of interventions commonly involving deprescribing. This service contributes significantly to the continuous optimisation of prescribing and monitoring of medicines. Medication optimisation reviews in residential and nursing homes therefore remain in the Medicines Management Team core agenda due to its established contribution to patient safety and impact on cost-effective prescribing over five years. In conclusion, the present research highlights that the work undertaken by primary case pharmacists and the use of standardied tools in Somerset for coding interventions and attributing safety scores are invaluable to collecting important data, and to build a clearer picture of deprescribing in the context of service cost-effectiveness.

## Figures and Tables

**Table 1:** Overall summary data for care home visits in Somerset in 2018–19.

GP Federation	Number of Care Homes	Number of Beds	Number of Registered Patients	Number of Care Home Visits	Number of Patient Reviews	Hours Spent on Review
GP Federation 1	19	580	442	8	106	65.95
GP Federation 2	22	495	412	17 (1st visit) + 3 (2nd visit)	347 (1st visit) + 46 (2nd visit)	373.75
GP Federation 3	36	1192	939	25 (1st visit) + 14 (2nd visit)	939 (1st visit) + 346 (2nd visit)	1016.45
GP Federation 4	37	810	578	8	181	108.50
GP Federation 5	38	1169	919	30	524	423.25
GP Federation 6	60	1792	1363	27	457	344.25
GP Federation 7	16	446	382	6	170	108.00
CCG Total	228	6484	5035	121 (1st visit) + 17 (2nd visit) = 138	2535 (1st visit) + 392 (2nd visit) = 2927	2440.15

**Table 2:** Overall summary data for interventions care home visits in Somerset in 2018–19.

**Intervention Type Totals**			
**Code Type**	**Number of Interventions** **(% of Total)**		**Potential Yearly Drug Saving (−) or Cost (+)**
1—Error Codes	490 (9.5%)		−£6037.47
2—Switch Codes	1038 (20.1%)		−£53,348.38
**3—Stop Codes (Deprescribing)**	**1264 (24.5%)**		**−** **£109,841.73**
4—Start Codes	86 (1.6%)		£1890.00
5—Allergy Codes	48 (0.9%)		n/a
6—Monitoring	717 (13.9%)		n/a
7—Miscellaneous Codes	1525 (29.5%)		−£44,761.93
**Total**	**5168**		**−** **£212,099.51**
**Safety Risk Assessment Scoring**			
**Risk level**		**Number of safety interventions** **(% of all safety interventions)**	
1—Minor		937 (82.0%)	
2—Moderate/Significant		191 (16.7%)	
3—Major/Serious		13 (1.1%)	
4—Catastrophic/Potentially Lethal		2 (0.2%)	
**Total**		**1143**	
**Nature of Deprescribing Interventions**			
**Deprescribing code**		**Number of deprescribing interventions** **(% of total deprescribing interventions; n = 1264)**	
3a—Stopped—clinical		269 (21.3)	
**3b—Stopped—no longer needed**		**750 (59.3%)**	
3c—Stopped—allergy/intolerance		4 (0.3%)	
3d—Stopped—C/I or interaction		3 (0.2%)	
3e—Stopped—side effects		14 (1.1%)	
3f—Stopped—duplication		112 (8.9%)	
3g—Stopped—sip feed		74 (5.9%)	
3h—Stopped—appliance		38 (3.0%)	

**Table 3:** NHS Somerset CCG examples of safety interventions review undertaken 03/01/2019.

Age of Patient (years)	Total Drugs on Medication Administration Record	Drug/Product/Appliance Name	Care Home Actions	Pharmacist/GP Practice Actions	Risk Safety Scoring	Comments & Outcomes from Safety Interventions	Risk Safety Scoring Review by Lead Pharmacists on 03/01/2019
94	10	On dual antiplatelets—discharge letter from 18 February. Had acute myocardial infarction—to remain on clopidogrel for 12 months only	Not applicable	Amend directions on clopidogrel to give a stop date of February 2019	1	94-year-old with history of falls could have been on dual antiplatelets indefinitely	1
102	12	Quinine bisulphate for leg cramps—no longer recommended interaction with furosemide + low estimated Glomerular Filtration Rate (eGFR)	Try without + monitor	Move to variable use repeat + reduce quantity	1	Quinine can be associated with thrombocytopenia	1
82	15	Discharged from hospital on quetiapine but seen 21.3.18 by Community Mental Health Team (CMHT) and this was stopped (still on repeat)	Do not administer	Discontinue quetiapine	1	Meds not aligned following CMHT review	2
89	9	On apixaban 2.5 mg twice daily (BD)—needs weight to determine if dose is ok	Weight patient	Weight = 64.4 kg + creatinine <133. Needs 5 mg BD	2	Currently on sub-therapeutic dose of Direct Oral Anticoagulant (DOAC)	2
96	9	Nitrofurantoin 50 mg tab 28 1on stopped. GP stopped and deemed replacement NOT required and monitor for symptoms of urinary tract infections (UTIs)	Amend records	Update patient medication record (PMR)	3	Risk of peripheral neuropathy. Antibacterial efficacy depends on renal secretion of drug into urinary tract. Avoid if eGFR < 45 ml/min/1.73 m^2^ as long term use. eGFR at review time 17. Therefore, this drug inappropriate and also appeared on Eclipse Red Alerts.	3
89	14	Apixaban 5 mg tabs—no obvious indication tasked X as concerned re dose-lady is <60 kg and over 80 years old.	GP error. This medication was not indicated for this lady and the dose was high for her age and weight.	GP informed of error and submitted critical incident form. Further error noticed 2/1/2019 as apixaban reissued after GP had stopped. Care home had reordered, and prescription clerk reissued - prescription stopped today before it had reached patient. Further incident added to critical incident at surgery.	3	Apixaban added to wrong records and prescription issued - given to patient for three months. 5 mg tablets given and patient over 80 years old and <60 kg could have been serious if no intervention. Critical incident error reported by X and Datix report by Y update 2/1/19 restarted by GP 24/12/18 care home had requested, and untrained person reissued. Stopped prescription before it got to care home 2/1/19	4 (“Drug related incident prevented which could have led to long-term patient harm or death of patient”)

**Table 4:** NHS Somerset CCG progress data summary of care homes reviews by financial year from April 2014–15 to March 2019.

Financial Year	Number of Care Homes Visits (% From Total)	Number of Patient Reviews	Total Number of Interventions (Number Per Patient Review)	Potential Annual Drugs Savings (Saving Per Patient Review)	Deprescribing (% From Total Savings)	Total safety Interventions (% From All Interventions)
2014–15	64	1195	2817	£106,762	£61,180	
	(27)		(2.3)	(£89)	(57)	
2015–16	62	1222	3631	£101,083	£62,117	660
	(27)		(3.0)	(£83)	(61)	(16.5)
2016–17	84	1622	4654	£122,479	£73,205	888
	(35)		(2.7)	(£75)	(60)	(19.1)
2017–18	151	3439	7685	£269,512	£125,149	1178
	(64)		(2.2)	(£78)	(46)	(15.3)
2018–19	138	2927	5168	£212,100	£109,842	1143
	(61)		(1.8)	(£72)	(52)	(22.1)
Total for 5-year period	499	10,405	23,955(2.3)	£812,441 (£78)	£431,493 (53)	3869 (16.1)

## Supplementary Materials

The following are available online at https://www.mdpi.com/2226-4787/7/3/105/s1, Figure S1: Care Home Visit Flowchart, Table S1: Care Home Data Collection Form, Table S2: Care Home Intervention Codes, Table S3: Care Home Safety Risk Assessment Scoring.
