# Supplementary material for: Deprescribing of Medicines in Care Homes—A Five-Year Evaluation of Primary Care Pharmacist Practices
Source: Pharmacy (Basel). 2019 Aug 3;7(3):105. doi: 10.3390/pharmacy7030105 (PMC6789793; doi:10.3390/pharmacy7030105)
Supplement: Supplementary file 1 [file pharmacy-07-00105-s001.zip › pharmacy-510002-supplementary/Figure s1 - Care Home Visit Flowchart.docx]

Agree service provision with GP practice including if GP (s) will take part in the visit with pharmacist, which homes should be prioritised, etc.

Telephone the Care Home Manager or Senior Nurse to arrange a suitable date and follow this with a formal confirmation letter with the GP Practice header. (This may vary according to internal agreement)

On the day of the visit or the preceding working day start filling in the relevant fields of the Somerset CCG Medicines Management team “Care Home Visit Data Collection” form.

Suggested ‘sub-searches’ following a main search for Residents in the Care Home include: antipsychotics, sip feeds, dressings, stoma and continence products. Relevant results can then be added as a note in “Care Home Visit Data Collection form” as a reminder during individual PMR reviews.

Review each PMR taking into account: all medicines and appliances, clinical appropriateness (and coding i.e. care home resident, dementia), acute/ repeat status, interactions, relevant monitoring (i.e. BP, TFT, LFT, U&Es, eGFR, DEXA, cholesterol, etc.), falls risk, allergies. (it may also be useful to collate a list of all current medicines)

During the visit fill in the relevant fields of the data collection form printout allocating one line per drug intervention. Keep the relevant staff member (s) contact (s) and agree Care Home visit follow up date.

Upon returning to the GP practice update the “Care Home Visit Data Collection form” electronically and add an administration note (i.e. review at the Care Home *with* on *date,* recommended interventions) to each PMR with the read code 8BIC - “Medication review done by pharmacist”. (To ensure confidentiality visits should be scheduled so you are able to return to the practice on the same day)

Send the updated form to GP (s) and once all actions are agreed and send appropriate final versions to: GP(s), relevant member of staff at the home, supplying pharmacy (ies) and CCG Medicines Management administrator and Locality Medicines Manager. (Ensure the appropriate level of confidentiality is maintained)

**Recommended resources:**

Care Quality Commission (CQC) Care Homes Webpage <http://www.cqc.org.uk/content/care-homes>

- CPPE distance learning programme “Care homes: supporting people, optimising medicines” – **mandatory**; available at <https://www.cppe.ac.uk/programmes/l/suppcarehome-e-00> . CPPE Care Homes Gateway <https://www.cppe.ac.uk/gateway/carehome>
- NHS Somerset CCG Prescribing resources <http://www.somersetccg.nhs.uk/about-us/how-we-do-things/prescribing-and-medicines-management/prescribing/>
- NICE Social care guideline “Managing medicines in care homes” March 2014 available at <http://www.nice.org.uk/guidance/sc1>; NICE Quality standard “Medicines management in care homes” March 2015 available at <https://www.nice.org.uk/guidance/qs85>
- RPS “Polypharmacy: Getting our medicines right” <https://www.rpharms.com/recognition/setting-professional-standards/polypharmacy-getting-our-medicines-right>
- SIGN Polypharmacy Guidance (also available as a mobile app) <http://www.polypharmacy.scot.nhs.uk/>
